# Two consecutive limb lengthenings with the same PRECICE nail: a technical note

**DOI:** 10.1007/s11751-018-0317-y

**Published:** 2018-09-19

**Authors:** André Couto, Joana Freitas, Nuno Alegrete, Jorge Coutinho, Gilberto Costa

**Affiliations:** Orthopaedic and Traumatology, Centro Hospitalar de São João, Alameda Prof. Hernâni Monteiro, 4200–319 Porto, Portugal

**Keywords:** Limb, Lengthening, Magnetic, Nail, Deformity

## Abstract

**Purpose:**

The most significant advance in our time about limb lengthening is the magnetic lengthening nail, as the first reports appeared to show good results with accurate lengthening rates and good regenerate bone formation. The described complication rate is generally low. They avoid external fixation elements, and are activated transcutaneously, so the patient’s pain and discomfort are reduced and the rehabilitation is faster and more effective. The aim of authors is to describe a special technical issue of the PRECICE system: the nail can be extended inside the patient limb (after the osteotomy), but it also can be retracted inside the limb after achieving the bone union.

**Methods:**

The authors present a case in which the limb lengthening has been performed in consecutive lengthening periods using the same nail. The nail was extended and retracted by altering the settings on the external remote control as well as accurately setting the rate of distraction.

**Results:**

After two consecutive femoral lengthening with the same PRECICE nail, the patient no longer has a significant lower limb length discrepancy and patient satisfaction was high. During this clinical case, we were not confronted with any type of complications.

**Conclusion:**

This technique utilizes the principles and advantages of lengthening over an magnetic lengthening nail, avoids the necessity of nail removal and minimizes the complication rates and the overall time for complete recovery.

**Level of evidence:**

Level IV.

## Introduction

Research and progress in medical devices have led to constant improvement in outcomes of limb lengthening [8]. Since the first lengthening performed at the beginning of the twentieth-century, many have contributed to the development of various limb lengthening techniques, using the principles of external fixation and lengthening nails [1].

Following the literature, the most significant advance in our time is the magnetic lengthening nail (PRECICE system), as the first reports appeared to show good results with accurate lengthening rates and good regenerate bone formation [3, 4, 7, 9]. They avoid external fixation elements, and are activated transcutaneously (without required rotation or other manipulations); the patient’s pain and discomfort are reduced, and they facilitate quicker and more effective rehabilitation [1, 2, 8].

The described complication rate is generally low, with the most common being implant failure to lengthen, nail breakage and premature consolidation; most are associated with poor implant choice (too short versus too long nail; small versus large nail diameter) and difficulty of placement of the external remote control (ERC) mainly in the proximal femur, because of the soft tissue deposit in obese patients [2–4, 7, 9].

The PRECICE nail is a magnet-operated telescopic internal lengthening device with an ERC that contains two rotating magnets: when placed by the patient on the skin, over the magnet within the nail, they cause this internal magnet to rotate which translates to the thinner nail element telescoping out of the thicker surrounding nail; the nail can be both extended and retracted by altering the settings on the ERC as well as accurately setting the rate of distraction; a distance of 1 mm requires the ERC to be placed over the magnet within the nail for 7 min [6]. The rate of distraction is the biggest point of discussion, depending on the lengthening localization (humerus, tibia, femur or even spine) and bone individual regenerate physiology.

The high price and short follow-up (< 5 years in most publications) is still an obstacle to its expansion as a gold standard technique for limb lengthening. However, reviewing the literature demonstrates that PRECICE is apparently more cost-effective compared to lengthening with external fixation [5, 10].

## Patients and methods

**Fig. 1:**
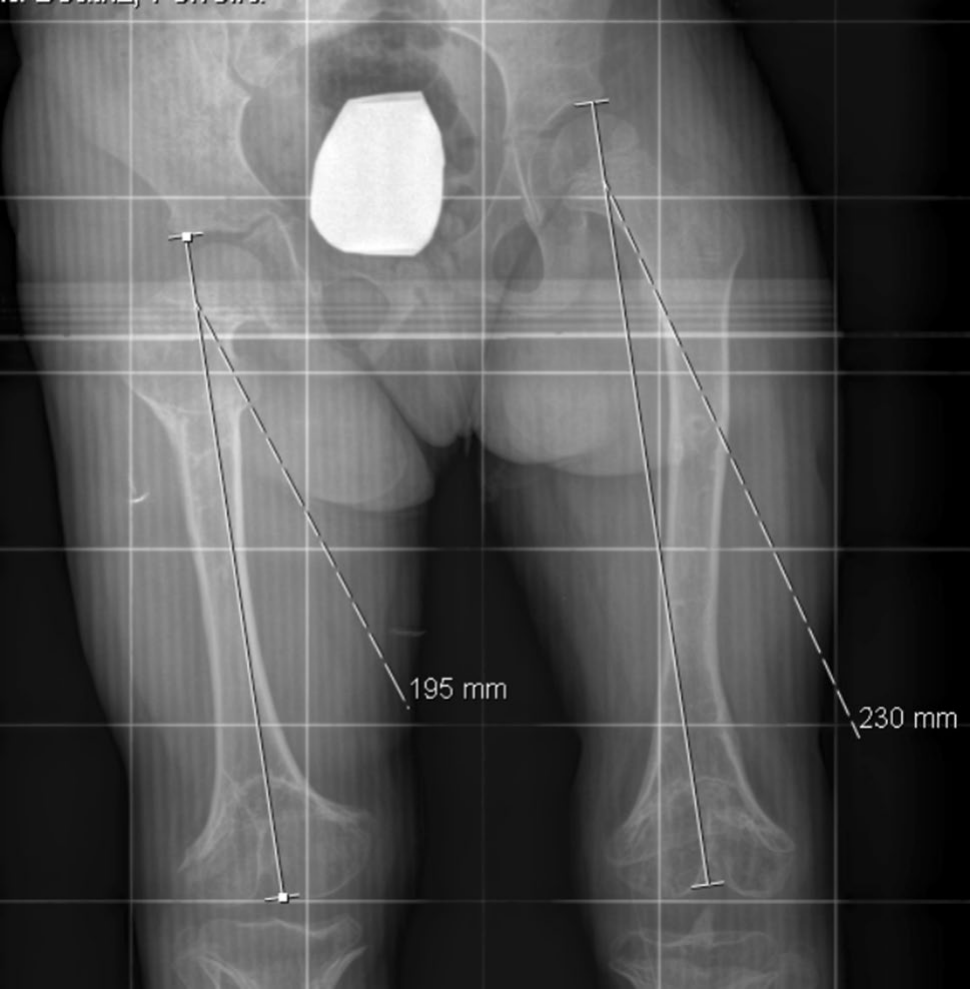
Preoperative X-ray with a right limb (femur) length discrepancy of 35 mm

A 9-year old girl was seen by our children’s orthopaedic department with a diagnosis of a right limb (femur) length discrepancy of 35 mm (sequelae of meningococcemia)—Fig. 1.

**Fig. 2:**
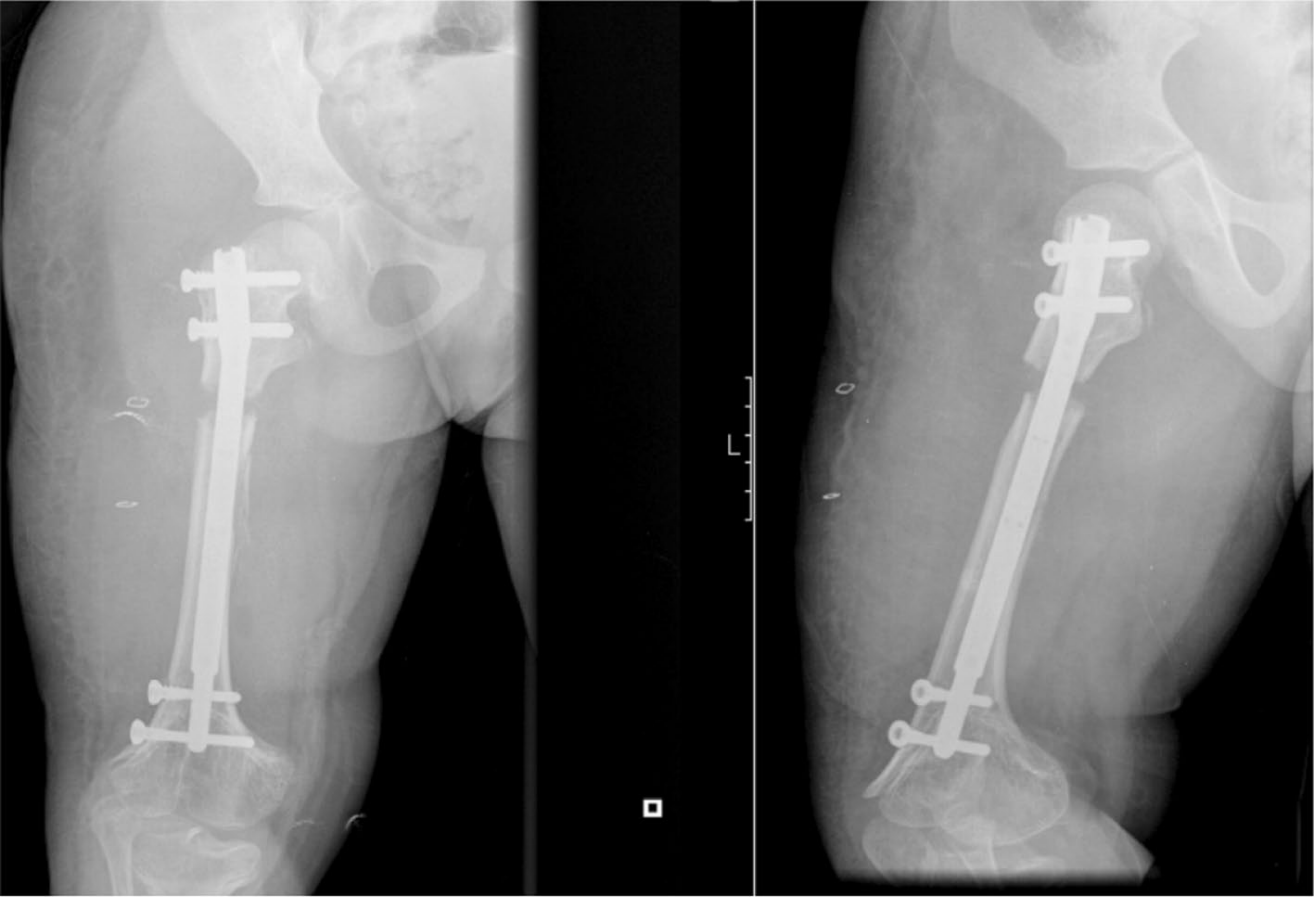
Two weeks postoperative

**Fig. 3:**
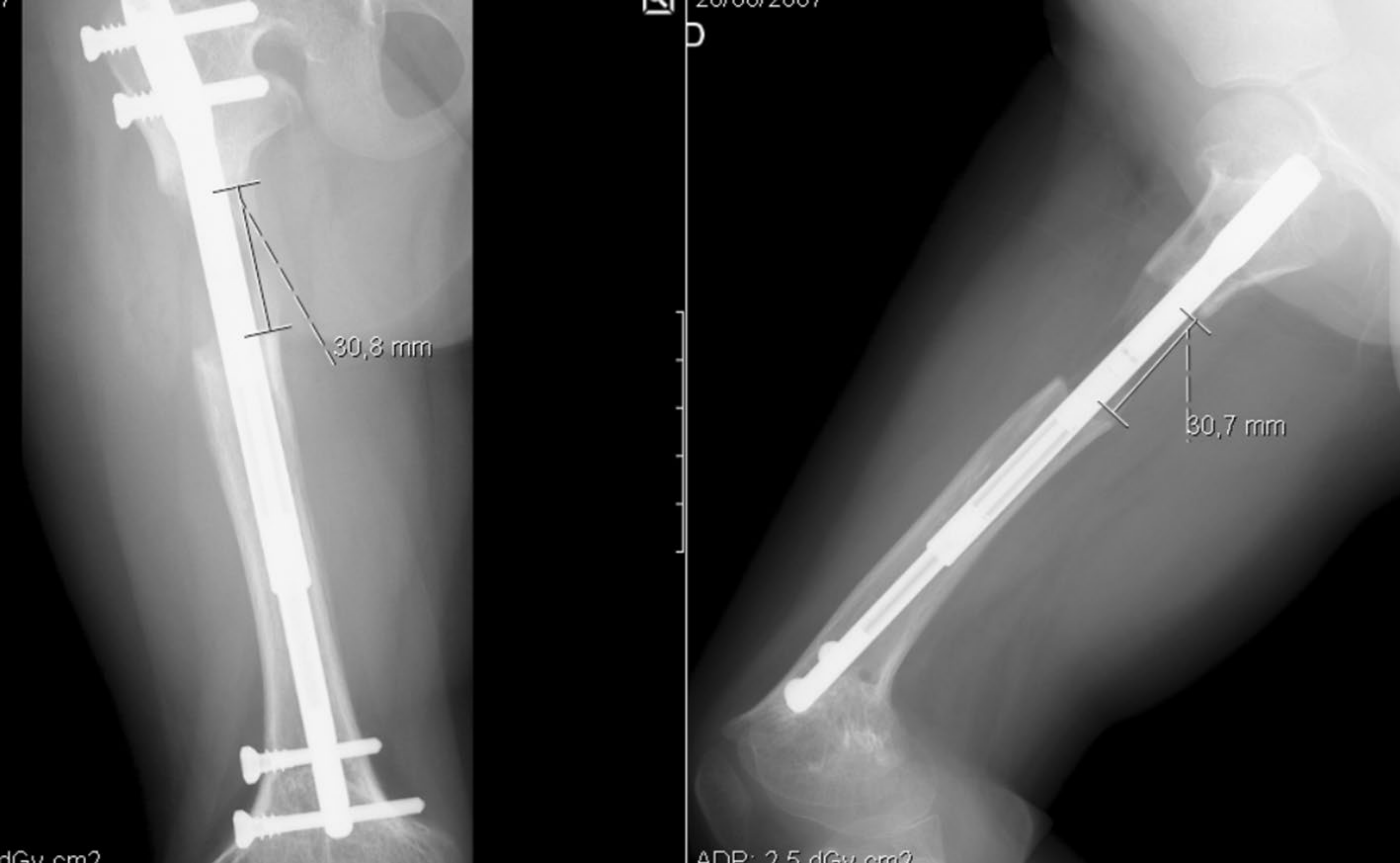
Two months postoperative

**Fig. 4:**
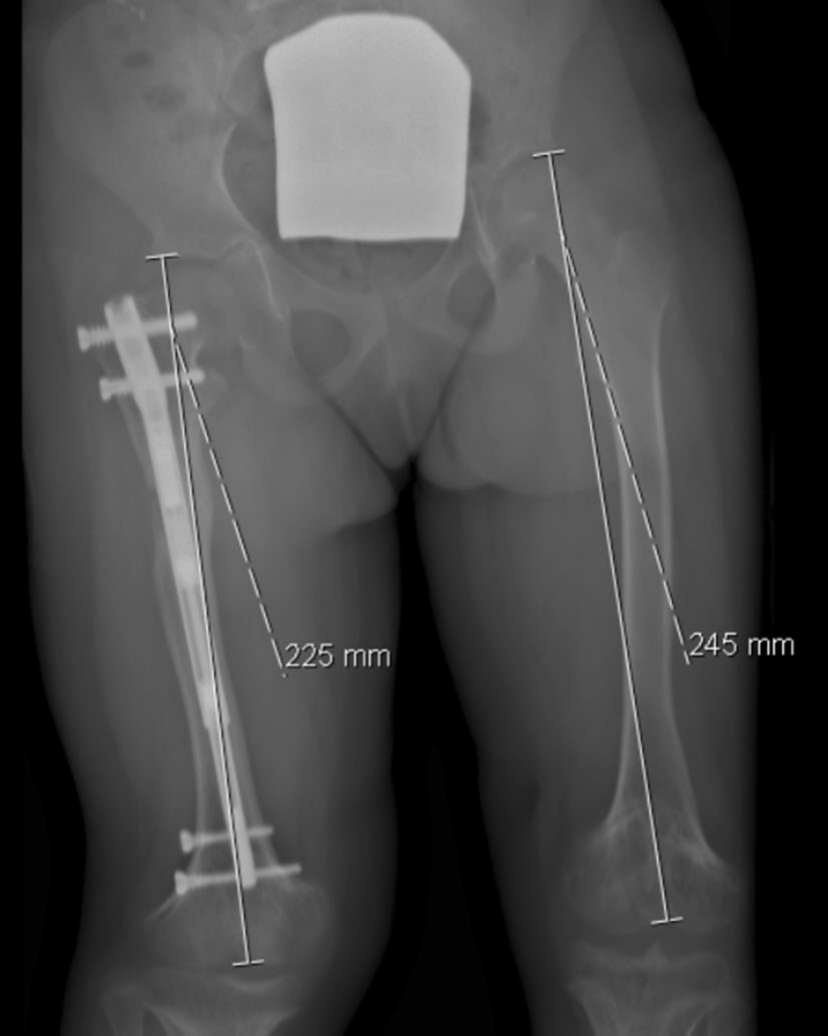
Six months postoperative

We performed a right femur lengthening with an intramedullary magnetic nail-PRECICE (Antegrade femur; diameter: 8.5 mm; telescoping rod: 30 mm; length: 170 mm). After implantation of the nail a first distraction of 1 mm was performed with the patient still under anesthetic. At 7 days after surgery, we started the distraction with a rate of 1 mm/day until we achieved the maximum of distraction of the nail telescoping rod (30 mm), occurring at 38 days after the first surgery. The patient was seen at different intervals with radiographs to monitor lengthening and bone union—Figs. 2, 3 and 4. After 6 months, radiographic bone union was achieved (corticalization in the regenerate bone was observed in at least three cortices). However, the patient still showed a right limb (femur) length discrepancy of 2.0 cm (as the left limb continued the normal growing process).

**Fig. 5:**
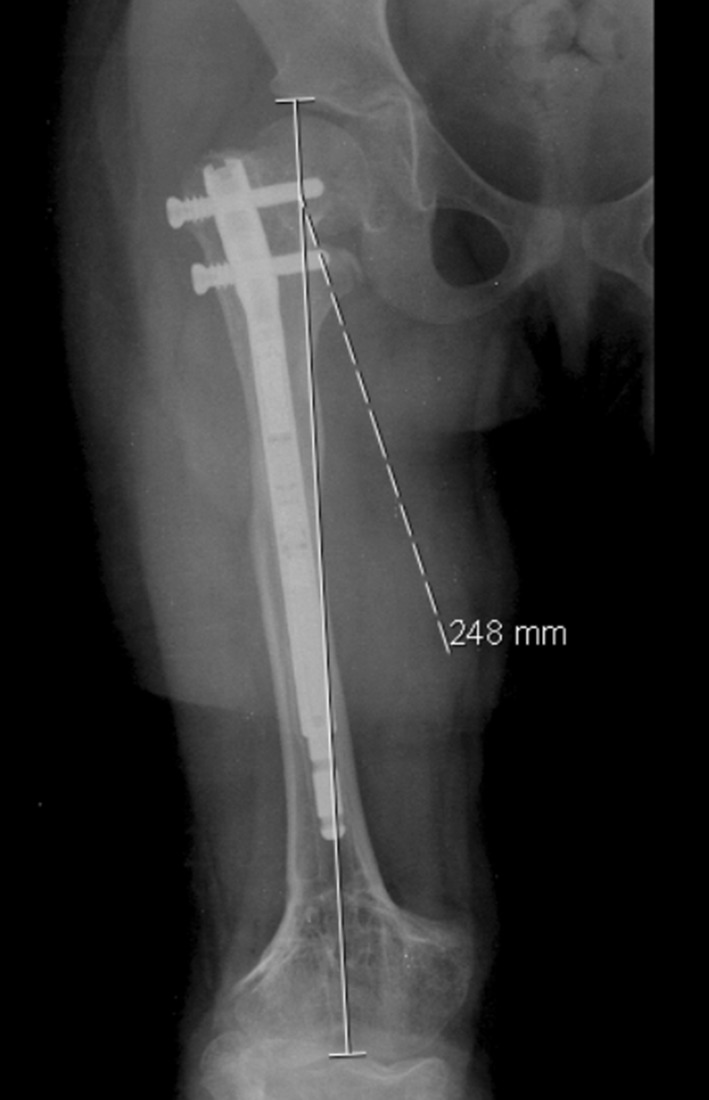
Postoperative X-ray after second surgery to remove distal locking screws

One year following the initial surgery, we performed a second surgery (in our outpatient department) to remove the distal locking screws—Fig. 5. After surgery, the patient performed retraction of the nail telescoping rod using the ERC (with an inverted program lasting 2 h).

**Fig. 6:**
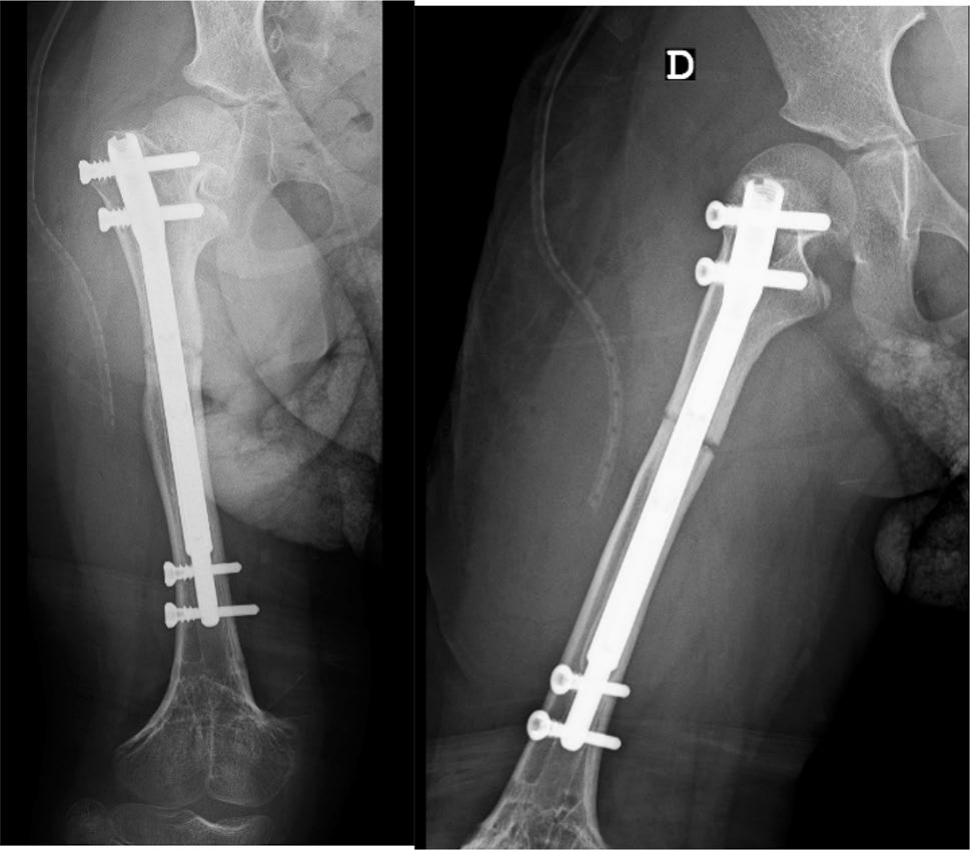
Postoperative X-ray after third surgery (new osteotomy and impute of distal locking screws)

**Fig. 7:**
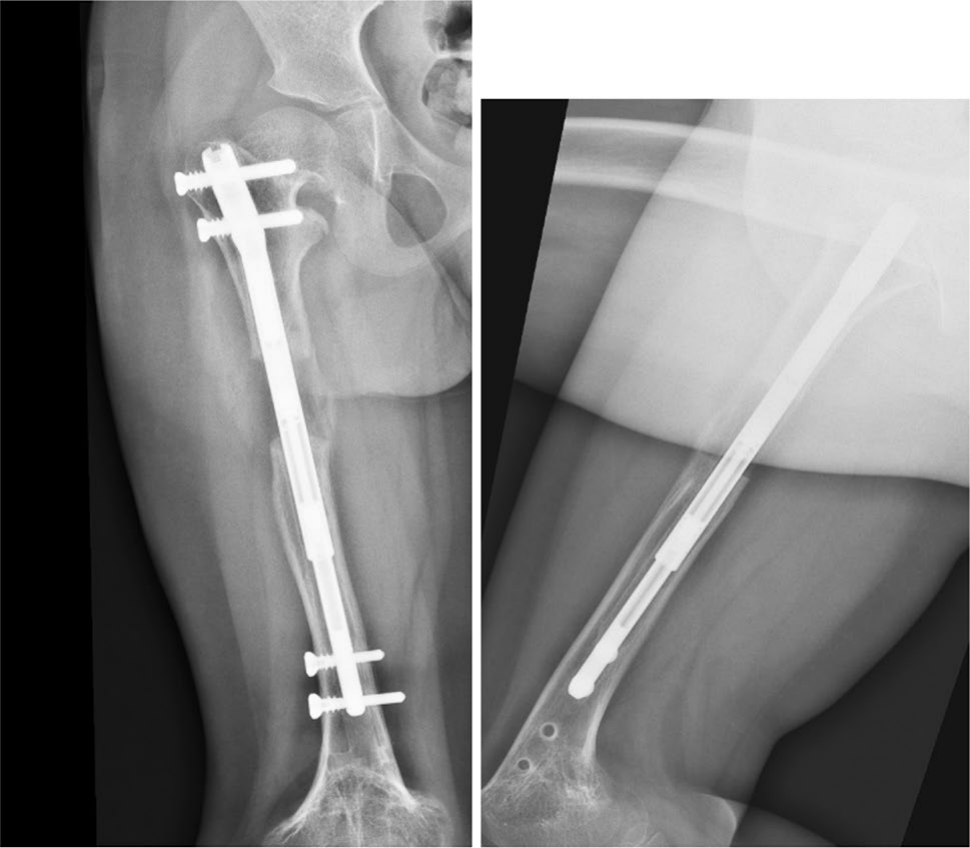
Two months postoperative

**Fig. 8:**
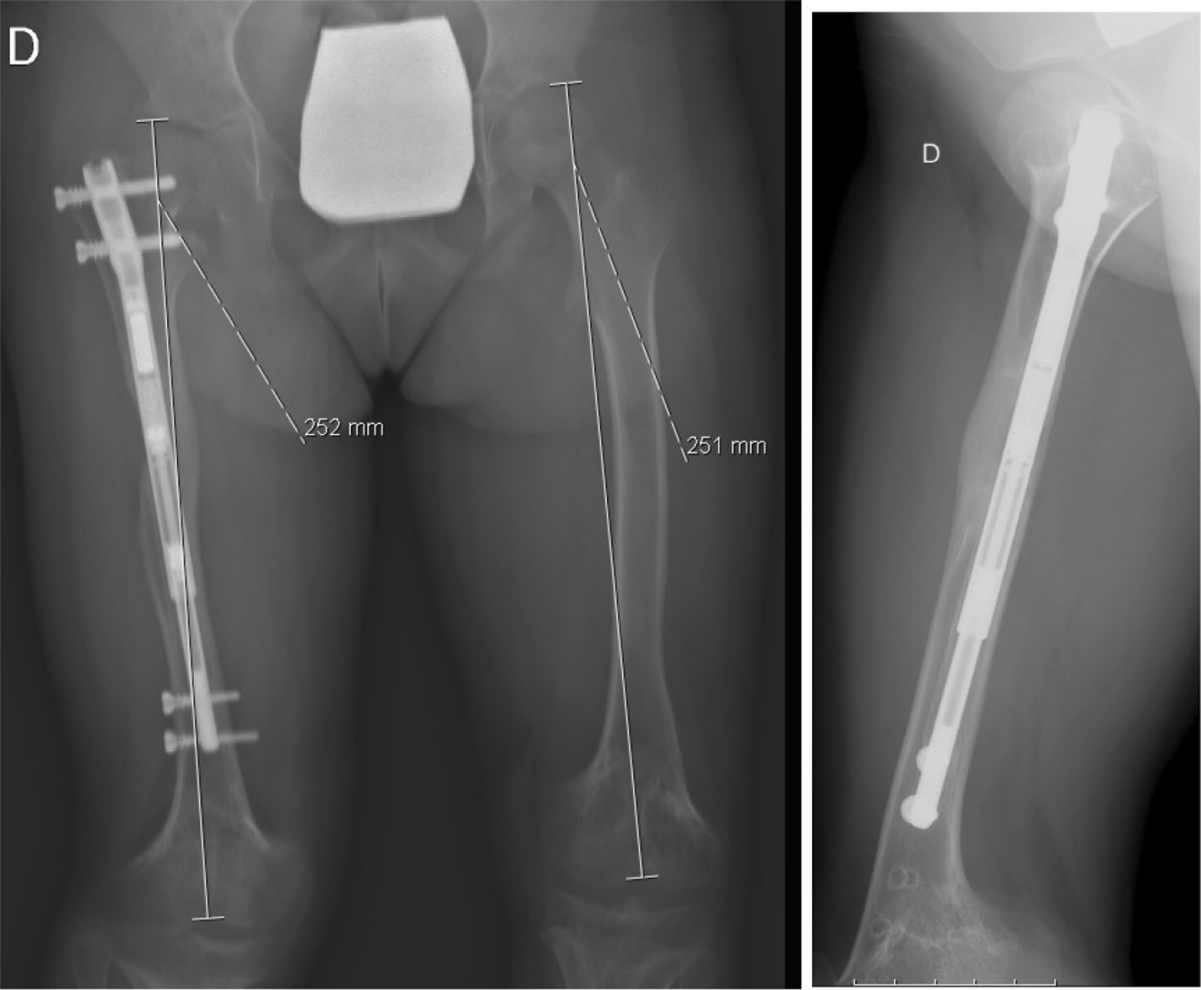
Six months postoperative

Three weeks later we performed a third surgery to impute new distal locking screws in the retracted nail; we also performed a new bone osteotomy (according to the localization of the nail magnet)—Fig. 6. We followed the same protocol: a first distraction of 1 mm was performed in the operating room; 7 days after surgery we restarted the distraction with a rate of 1 mm/day until we achieved the limb distraction necessary (30 mm). The patient was seen at different intervals with radiographs to monitor lengthening and bone union—Figs. 6, 7 and 8. After almost 6 months, we obtained radiographic bone union, this time without lower limb length discrepancy.

## Results

After two consecutive limb (femoral) lengthening operations with the same PRECICE nail, the patient no longer has a significant lower limb length discrepancy. The patient and parent’s global satisfaction with the final the result was high.

As the patient was 12 years old at the end of the treatment, she remains under follow-up at our department, at standard time intervals with lower limb radiographs to monitor the lower limb growth and prevent a possible new length discrepancy.

## Discussion

One thing that becomes clear when reviewing the available literature addressing limb lengthening is that we have witnessed extraordinary advances in modern times, since the first description of the Ilizarov method, passing the sophisticated circular fixators and software programs to gradually correct complex deformity (Hexapod) and the introduction of reliable magnetic intramedullary lengthening nails [1].

The aim of this paper is to describe a special technical issue of the PRECICE system: the nail can be extended inside the patient limb (after the osteotomy), but it also can be retracted inside the limb after achieving the bone union (we just need to remove the distal screws and alter the settings on the ERC). During this clinical case, we were not confronted with any type of minor or major complication associated with this procedure.

We believe this clinical case is the first being described in which the limb (femoral) lengthening has been performed in a consecutive lengthening period using the same PRECICE system nail and obtaining a good final outcome.

As referred to before, the PRECICE nail is a magnet-operated telescopic internal lengthening device with an ERC that contains two rotating magnets: when placed by the patient on the skin, over the magnet within the nail, they cause this internal magnet to rotate which translates to the thinner nail element telescoping out of the thicker surrounding nail. However, the nail can be both extended and retracted by altering the settings on the ERC as well as accurately setting the rate of distraction.

While recent research demonstrates that patients prefer PRECICE to external fixation [3, 4, 7, 9], it is important for healthcare facilities to examine the clinical and economic implications associated: literature analysis demonstrates that PRECICE is apparently a cost-saving alternative to external fixation due to the lower rates of surgical complications and shorter inpatient hospital stays associated with the procedure [5, 10]. Although we are unable to provide a direct comparison with other lengthening technics, we can speculate that some of the increased costs of PRECICE are mitigated by fewer complications, fewer re-operations, and shorter rehabilitation time. Saying this, an accurate analysis is needed to assess the real cost-effectiveness comparing with external fixation lengthening option.

The bone healing assessment on plain X-ray is highly subjective, with wide inter- and intra-observer variation; the bone mineralization is better assessed in terms of pixel value ratio (PVR: ratio of pixel value of regenerate to adjacent bone) on picture archiving and communication system (PACS) digitized radiographs, providing objective assessment of callus formation [8]. Notwithstanding this question, our clinical option to confirm the bone union was based on the authors’ experience in X-ray visual confirmation (presence of three or more cortices in the bone lengthening area), which could be described as a limitation of this paper.

## Conclusion

The intramedullary magnetic nails seem to decrease patients’ pain and discomfort, while facilitating more rapid and effective rehabilitation, when compared with conventional techniques using external fixation. One of the obvious advantages is the ERC, which allows transcutaneous activation of the growing nail; however, it also allows, if necessary, the programming of a retrograde distraction to retract the nail telescoping rod, improving our clinical and technical options to treat the limb length discrepancy.

After a literature review, we believe this clinical case is the first described in which the limb (femoral) lengthening has been performed in consecutive lengthening periods using the same PRECICE system nail, with a good clinical outcome and high patient satisfaction.

Notwithstanding the clinical advantages already described in the literature, in the authors’ opinion, this technical setting is another positive variable that can help to confirm the use of magnetic growing nails as state-of-the-art and the gold standard technique in limb lengthening.
